# Diagnosis and Management of Cancers in Pregnancy: The Results of a Dual Battle Between Oncological Condition and Maternal Environment—Literature Review

**DOI:** 10.3390/cancers17030389

**Published:** 2025-01-24

**Authors:** Mihai-Daniel Dinu, Romina-Marina Sima, Andrei-Sebastian Diaconescu, Mircea-Octavian Poenaru, Gabriel-Petre Gorecki, Mihaela Amza, Mihai Popescu, Mihai-Teodor Georgescu, Ancuta-Alina Constantin, Mara-Madalina Mihai, Cristian-Valentin Toma, Liana Ples

**Affiliations:** 1 Department PhD, IOSUD—Institution Offering Doctoral Studies, “Carol Davila” University of Medicine and Pharmacy, 020021 Bucharest, Romania; mihai-daniel.dinu@drd.umfcd.ro (M.-D.D.); mihaela.amza@umfcd.ro (M.A.); 2 Department of Obstetrics and Gynecology, “Carol Davila” University of Medicine and Pharmacy, 020021 Bucharest, Romania; romina.sima@umfcd.ro (R.-M.S.); mircea.poenaru@umfcd.ro (M.-O.P.); 3 “Bucur” Maternity, Saint John Hospital, 012361 Bucharest, Romania; 4 Faculty of Medicine, “Carol Davila” University of Medicine and Pharmacy, 020021 Bucharest, Romania; mihai.georgescu@umfcd.ro (M.-T.G.); mara.mihai@umfcd.ro (M.-M.M.); cristian.toma@umfcd.ro (C.-V.T.); 5 General Surgery Department, Fundeni Clinical Institute, 022328 Bucharest, Romania; 6 Department of Anesthesia and Intensive Care, Faculty of Medicine, “Titu Maiorescu” University, 031593 Bucharest, Romania; gabriel.gorecki@prof.utm.ro; 7 Department of Anesthesia and Intensive Care, CF2 Clinical Hospital, 011464 Bucharest, Romania; 8 Department of Anesthesia and Intensive Care, “Carol Davila” University of Medicine and Pharmacy, 020021 Bucharest, Romania; mihai.popescu@umfcd.ro; 9 Bucharest University Emergency Hospital, 169 Splaiul Independentei, 050098 Bucharest, Romania; 10 “Prof. Dr. Al. Trestioreanu” Oncology Discipline, “Carol Davila” University of Medicine and Pharmacy, 020021 Bucharest, Romania; 11 Department of Cardio-Thoracic Pathology, “Carol Davila” University of Medicine and Pharmacy, 020021 Bucharest, Romania; ancuta-alina.constantin@umfcd.ro; 12 Institute of Pneumology “Marius Nasta”, 050159 Bucharest, Romania; 13 Department of Oncologic Dermathology, “Elias” University Emergency Hospital, 010024 Bucharest, Romania; 14 “Prof. Dr. Theodol Burghele” Clinical Hospital, 061344 Bucharest, Romania

**Keywords:** cancer, pregnancy, fetus, radiological diagnosis, nuclear medicine, chemotherapy, radiotherapy, surgery, targeted therapy, immunotherapy, breastfeeding, fertility preservation, psychological effects

## Abstract

Cancer during pregnancy presents considerable challenges that arise from various factors, including the diagnostic, prognostic and therapeutic implications of managing cancer in this unique patient population. There is a crucial need for an integrated approach that aligns medical interventions for both the pregnant patient and the developing embryo or fetus. Furthermore, the distinct characteristics associated with each stage of gestation may significantly influence the treatment protocols that can be proposed. Due to all of these factors, the importance of collaboration among healthcare professionals from different specialties (to ensure that both mother and child receive optimal care throughout the pregnancy) is often neglected. This review is designed to provide a thorough overview of the current standard procedures regarding the diagnosis and treatment options for cancer in pregnant patients in order to ensure the safety of mother and child. Furthermore, the review describes the feasibility of current fertility preservation methods, highlights the psychological effects of cancer during pregnancy and examines the risks and benefits of breastfeeding.

## Simple Summary

Cancer during pregnancy presents unique challenges in diagnosis and treatment. This study discusses the best management approaches, emphasizing the importance of full-term delivery when possible. It also highlights safe diagnostic and therapeutic strategies, as well as post-birth considerations for both mother and child. While breastfeeding may not always be advisable, it should not be excluded in every case, and careful evaluation is required to determine its safety based on treatment.

## 1. Introduction

Gestational cancer refers to cancer that develops during pregnancy or within the year following childbirth. The incidence among European countries has shown relative stability in the twenty-first century, particularly after experiencing a consistent annual increase of 1.5% during the last decades of the previous century. Even so, it is still considered a rare condition. Currently, the incidence rate is estimated to be 1 case per 1000 pregnancies [1]. Factors like shifts in societal and demographic patterns (such as women opting to have children later in life, resulting in higher maternal age) [2] contribute to the need for a more detailed analysis of this pathology. Furthermore, advanced prenatal screening methods like noninvasive prenatal testing (NIPT) for detecting fetal aneuploidies can sometimes uncover hidden cancers in pregnant women by identifying unusual genome patterns, including somatic copy number changes [3].

A diagnosis of cancer during pregnancy might pose significant challenges in terms of treatment and survival [4]. Moreover, it can lead to adverse obstetric and neonatal outcomes. According to the literature, nearly 15% of patients with a diagnosis of pregnancy-associated cancer experience a wide range of complications, such as premature rupture of membranes before week 37, intrauterine growth restriction, blood loss and preeclampsia. However, this risk is not considered to be significantly higher than that observed in the population of pregnant women without cancer [5]. Furthermore, the rate of spontaneous preterm birth is only slightly higher than that reported in the general population [6]. Medically induced labor and elective cesarean sections are more common in pregnant women with cancer, as early pregnancy termination is usually necessary to enable pharmacological or surgical treatment [5,6].

Breast cancer is the most frequently occurring malignancy during pregnancy (accounting for one-third of all reported cases), with an incidence rate of 1 in 3000 pregnancies [7]. Other malignant conditions with lower incidence include cervical cancer, hematological malignancies, ovarian cancer, colorectal cancer and melanoma [8,9].

When there is suspicion of cancer, diagnostic procedures must be promptly initiated. Once the diagnosis is confirmed, pregnant patients should receive care from a multidisciplinary team. A high level of collaboration between different medical specialties is vital, beginning with accurate diagnosis and continuous monitoring of both mother and fetus and extending throughout the follow-up period. This coordinated approach is essential for ensuring the best possible care for the expecting parents and the unborn child, by carefully balancing the risks and benefits to both the mother and the fetus. Furthermore, medical decisions, along with counseling for the pregnant woman and her partner, should be guided by ethical principles.

The diagnosis of cancer during pregnancy presents a highly complex and challenging situation for the mother-to-be, her unborn child and the medical team responsible for their care. The quantity of evidence-based information regarding diagnostic methods, treatment options and maternal outcomes remains quite limited, particularly in relation to the extensive literature available for patients with malignant conditions who are not pregnant [10]. However, recent advancements and evidence-based findings related to cancer management in pregnancy, as well as numerous international expert consensus meetings, have contributed to a more standardized approach aimed at ensuring optimal outcomes for both mother and child [2,10]. Consequently, the management strategies for pregnant women diagnosed with cancer have seen significant improvements over the years. Presently, oncological therapies are preferred over the option of terminating a pregnancy. An international cohort study that examined more than 1000 patients demonstrated a trend toward an increasing number of live births over time. Nevertheless, it is important to recognize that preterm delivery is still a major risk factor for unfavorable neonatal outcomes in pregnancies complicated by cancer, and measures should be implemented to lessen this risk [2,11].

## 2. Diagnosis of Cancer During Pregnancy

### 2.1. Clinical Assessment and Diagnostic Investigations

The time from cancer diagnosis to the start of treatment may be delayed in pregnant patients due to both the psychological and physiological changes associated with pregnancy, along with multiple symptoms [12]. Therefore, during the clinical examination, symptoms such as vaginal bleeding, post-coital bleeding, anemia, fatigue, discharge and breast tenderness or lumps should be carefully evaluated [5,13]. Furthermore, a detailed medical history and clinical examination must be performed at the beginning of each pregnancy to identify any predisposing genetic factors (such as BRCA1/BRCA2 mutation carrier status or a strong family history of breast or ovarian cancer) or other malignancy risk factors [14]. A thorough assessment of new signs or symptoms should be routinely performed before attributing them to normal pregnancy-related changes. If certain symptoms raise suspicion for cancer, a complete physical examination (including the genitals, uterus, breasts, peripheral lymph nodes and skin) should be conducted [14].

### 2.2. Radiological Diagnosis

While it is legitimate to seek to minimize radiation exposure during pregnancy, it is also essential to acknowledge that the consequences of a missed or delayed diagnosis can pose a greater danger to both the mother and the fetus than the risks associated with ionizing radiation.

Table 1 summarizes information about the imaging techniques used during pregnancy, as well as about the contrast agents.

The application of ionizing radiation in medical imaging, such as X-rays and computed tomography (CT) scans, can lead to biological changes, but these effects are contingent upon both the gestational stage of pregnancy and the dosage of radiation used.

During the earliest phase of pregnancy, specifically within the first 10 to 14 days post-conception, the primary theoretical concern is the risk of spontaneous miscarriage. However, no evidence has shown this risk to manifest with the radiation levels commonly employed in routine diagnostic procedures, where doses are typically lower than 1 milligray (mGy). As pregnancy progresses into the second to fifteenth week, there is a heightened focus on limiting radiation exposure, particularly because of the sensitive developmental period of the embryo. Nevertheless, diagnostic imaging that targets areas outside of the abdominal region, such as imaging of the head, neck, thorax and extremities, does not present a significant risk to the fetus. In these cases, any radiation exposure occurs via scattered radiation (which is minimal and often negligible when standard safety protocols are applied). Shielding and other precautionary measures ensure that the scattered radiation is so low that it presents no substantial risk to fetal health [17,18].

In situations where imaging of the abdominal or pelvic area is necessary, the procedure can be adjusted to optimize safety. In this way, the radiation dose absorbed by the fetus remains far below any level that could induce developmental abnormalities. Current research indicates that fetal exposure to radiation below 100 millisieverts (mSv) does not result in detectable harm, and as such, there is no medical justification for considering termination of pregnancy at these levels [22]. For radiation exposure levels exceeding 100 mSv, the likelihood of fetal malformations remains minimal. However, radiation doses that surpass 150 mSv are linked with an increased risk of developmental abnormalities (particularly during the critical periods of organ formation). After the fifteenth week of pregnancy, concerns about developmental issues largely subside, with the primary risk shifting to the potential for radiation-induced cancer in later life. This risk is only considered significant if radiation exposure exceeds 150 mSv.

It is generally accepted that fetal radiation doses of up to 1 mGy are well within safe limits, as this is comparable to the natural background radiation that the fetus is typically exposed to in the environment [23,24]. Thus, in most clinical settings, the minimal radiation exposure associated with diagnostic imaging, when managed carefully, does not pose a substantial threat to the health and development of the fetus.

During the lactation phase, the administration of iodinated or gadolinium-based contrast agents is generally considered to pose minimal risk to breastfeeding infants. This is largely due to the very low concentrations of these substances that are transferred through breast milk, specifically about 0.01% of the dose that the mother receives for gadolinium and approximately 0.5% for iodine. Furthermore, only a small percentage of the contrast that enters the infant’s gastrointestinal tract is absorbed, which further mitigates potential risks. Research indicates that the peak concentration of contrast agents in breast milk occurs roughly five hours after administration and becomes undetectable after twelve hours. Consequently, breastfeeding may be continued after the use of these agents. However, if mothers prefer to avoid exposing their infants to these contrast agents after being adequately informed, it is advisable for them to discard breast milk for a duration of 24 h following the injection. Additionally, special consideration must be given to premature infants, as their thyroid regulation systems may not be fully matured. This immaturity raises concerns about the risk of developing transient hypothyroidism [19].

### 2.3. Nuclear Medicine in Cancer Diagnosis

Sentinel lymph node biopsy (SLNB) is recognized as a standard technique for staging breast cancer. However, due to a lack of available data in the literature, the scientific evidence supporting the use of SLNB in the context of breast cancer during pregnancy is primarily derived from cohort studies [25]. This means that there is a notable absence of level 1 evidence, resulting in recommendations from various guidelines being classified at lower levels, specifically C or D. According to the National Comprehensive Cancer Network (NCCN) Clinical Practice Guidelines in Oncology, decisions regarding the use of SLNB should be made according to the individual circumstances of each patient [26]. Moreover, several other guidelines recommend SLNB when both axillary ultrasound and biopsy results for suspicious lymph nodes yield negative findings [27].

Lymphoscintigraphy and SLNB using 99mTc-albumin nanocolloids do not result in significant uterine irradiation when optimized protocols are adhered to [28,29]. Scheduling lymphoscintigraphy as the first procedure of the day to avoid contact with other nuclear medicine patients, as well as decreasing the time interval between the injection of nanocolloids and the surgical procedure, represents an essential action to minimize fetal exposure. Consequently, when recommended as a one-day protocol, radiocolloids are deemed safe [26,27]. According to a retrospective study, the procedure demonstrated high efficacy, with an identification rate of 99.3% and a very low rate of axillary recurrence at 0.7%. Therefore, there is no justification for contraindicating SLNB during pregnancy [25].

Concerning SLNB for melanoma, research findings have revealed considerable inconsistency in its effectiveness when performed during pregnancy [30]. Despite this, SLNB utilizing radiocolloids can be safely recommended following a thorough individual risk evaluation [31]. The general agreement among experts is that this procedure should not be conducted before the second trimester. Furthermore, it should follow a streamlined protocol carried out within a single day, utilizing 10–25 MBq of 99mTc, with the surgical intervention scheduled immediately after the completion of lymphatic mapping [32,33]. This approach ensures careful monitoring and optimal timing to enhance both maternal and fetal safety.

The ESMO guidelines advise against the use of bone scintigraphy and positron emission tomography (PET) during pregnancy due to potential risks associated with radiation exposure to the developing fetus [8]. The uncertainty surrounding the long-term effects of radiation on fetal development underscores the need for caution and alternative diagnostic approaches during pregnancy, ensuring both maternal and fetal safety remain a priority in clinical practice.

Certain evidence from a handful of limited studies suggests that the radiation dose to the fetus resulting from fluorodeoxyglucose (18F-FDG) positron emission tomography (PET) and PET/magnetic resonance imaging (MRI) is minimal, particularly during the later trimesters of pregnancy [34]. Despite this indication of lower exposure, the available data are inadequate to formulate a definitive recommendation for utilizing PET in cancer staging during pregnancy. This cautious approach stems from the need to prioritize fetal safety, as the long-term effects of any radiation exposure on fetal development remain uncertain. Thus, while these imaging modalities may present lower risks, the lack of comprehensive studies restricts their endorsement in clinical practice for pregnant patients with cancer.

## 3. Therapeutic Approaches and Timing for Cancer Management During Pregnancy

### 3.1. Surgery

Surgical interventions are considered to be safe across all trimesters of pregnancy. Nevertheless, the early second trimester is often preferred for surgical procedures (especially those involving intra-abdominal access). In this way, the uterus has not yet increased significantly in size, and at the same time, the risk of miscarriage is reduced [35]. Furthermore, findings have also indicated a correlation between surgical procedures performed during the early weeks of pregnancy, particularly between weeks 3 and 5, and a heightened risk of neural tube defects [10]. A higher chance of preterm delivery was also observed during surgical interventions in pregnancy [36]. As a result, the pregnant patient should be informed about the risk. Additionally, healthcare providers should take into consideration different strategies to reduce fetal morbidity once the fetus reaches viability (for example, the administration of corticosteroids to facilitate lung maturation in preparation for potential early delivery). Whenever the situation allows, the use of locoregional anesthesia is prioritized over general anesthesia due to its safety and efficacy benefits [37].

When deciding whether to utilize a laparoscopic method or a laparotomy, various considerations come into play. These include the gestational age of the patient, the surgeon’s expertise and familiarity with the procedure, the projected length of the surgery—which should preferably not exceed 90 to 120 min—and the capacity to sustain a low intra-abdominal pressure, ideally maintained within the range of 10 to 13 mmHg [35]. A laparoscopic procedure is generally viewed as a practical and secure option for pregnant patients, with its use deemed appropriate until approximately the 26th to 28th week of pregnancy. This time frame allows for the benefits of minimally invasive surgery without compromising maternal or fetal safety [10].

Intraoperative fetal heart rate (FHR) monitoring after viability should be coordinated with the patient and the surgical, obstetric and neonatology teams to outline the appropriate responses in the event of non-reassuring FHR patterns during the procedure. Additionally, utilizing postoperative fetal Doppler monitoring may be advantageous. In cases where uterine manipulation occurs during surgery, the administration of tocolytics may be warranted for up to 48 h postoperatively to minimize the risk of uterine contractions [38,39].

Due to the restricted range of analgesic options that are safe for use during pregnancy, it is strongly recommended to engage with the anesthesia team or any specialized acute pain management service that may be accessible. The expertise of these professionals can help adjust pain management strategies while considering the unique challenges associated with analgesia in this population. For example, in the recovery phase following surgery, various pain relief and anti-nausea medications can be administered. Commonly prescribed analgesics include paracetamol, tramadol, morphine and non-steroidal anti-inflammatory drugs (NSAIDs). For the management of nausea and vomiting, anti-emetic agents like metoclopramide or ondansetron are often recommended. However, particular caution must be exercised with NSAIDs during the third trimester of pregnancy, as their use has been associated with a significant risk—reported in 50–80% of cases—of premature closure of the ductus arteriosus, potentially leading to pulmonary hypertension in the newborn [10].

Surgery, immobility, malignancy and pregnancy together create a highly prothrombotic state that significantly increases the risk of thromboembolic events. Consequently, it becomes essential to implement strategies for preventing venous thromboembolism (VTE). Low molecular weight heparin has a well-established role in reducing the incidence of thromboembolic complications [38]. Additionally, using an intermittent pneumatic compression device may provide valuable mechanical support to enhance venous return and may further diminish the likelihood of VTE.

#### 3.1.1. Breast Cancer Surgery

Women diagnosed with breast cancer often require consultation to address fertility preservation and also to develop a plan for pregnancy and lactation [40,41]. A common symptom of breast cancer in pregnancy is a painful lump. Due to physiological breast changes during pregnancy, such as nipple discharge, hypertrophy and engorgement, breast cancer is often diagnosed at a more advanced stage [10,42]. Breast ultrasonography is considered the gold standard for diagnosis during pregnancy, offering high sensitivity and specificity for differentiating between solid and cystic masses and for lymph node evaluation [40]. Mammography may also be performed when necessary, with appropriate protective measures such as abdominal shielding, and histological confirmation can be obtained through core biopsy under local anesthesia. To assess for metastases, liver ultrasound, chest X-ray and skeletal MRI without contrast are recommended [10,42].

The surgical management of breast cancer during pregnancy is a complex and challenging undertaking, with the available data primarily derived from retrospective studies and individual case reports. Historically, there was a misguided belief that terminating a pregnancy might enhance a patient’s prognosis, but this assumption has been discredited by contemporary research. At this moment, it is widely accepted that surgical treatment for pregnant women with breast cancer follows an approach similar to that of non-pregnant patients. Depending on the cancer’s stage and extent, surgical options include either lumpectomy or mastectomy. These procedures are generally considered safe for the fetus and can be carried out during any trimester of pregnancy. On the other hand, certain aspects of breast cancer treatment (such as breast reconstruction) should be postponed until after the baby is delivered. Moreover, it is also recommended that radiation therapy following either mastectomy or lumpectomy be delayed until the pregnancy is over [43,44].

#### 3.1.2. Cervical Cancer Surgery

Cervical carcinoma, while the most common gynecologic malignancy associated with pregnancy, is relatively rare, occurring in approximately 1 in 1200 to 10,000 pregnancies [45].

Conservative surgical treatment of cervical cancer can be performed by laparotomy or laparoscopy. However, laparotomy is generally safe only up to 14–16 weeks of gestation. Current data suggest that simple trachelectomy is considered safe in contrast to radical trachelectomy [35]. Furthermore, lymphadenectomy is feasible up to 24 weeks of gestation. Although there is an increased risk of bleeding, which becomes more pronounced as pregnancy progresses, conization alone is considered sufficient and safe during pregnancy for stage IA or IA1 of cervical cancer diagnosed before 22–25 weeks of gestation. In these cases, a simple trachelectomy is a safe option [35].

For stage IA2, IB1, IB2 or IIA cervical cancer, if the pregnancy is below 22 weeks, lymphadenectomy is chosen as the first option. Moreover, in cases of stage IIIC cervical cancer (with lymph node involvement confirmed by lymphadenectomy), where the pregnancy is continued, chemotherapy with cisplatin should be initiated. Although combinations of paclitaxel with platinum salts are also considered safe, cisplatin monotherapy remains the preferred regimen [46,47]. It is important to note that radiotherapy is not an option for this type of cancer if the mother expresses an intention to continue the pregnancy. Furthermore, lymphadenectomy is not necessary in stages IB2 and IIA when there is no involvement of the lymph nodes, and chemotherapy may be chosen.

A general consensus is that for tumors under 2 cm identified in the third trimester of pregnancy, maternal and fetal monitoring until fetal maturity (followed by surgical intervention) may be considered. Chemotherapy should be administered in locally advanced stages (IB3-IIA2-IIB). For cervical cancer, cisplatin is the preferred chemotherapeutic agent, as it can be safely administered (except during the first trimester of pregnancy, when it may harm the fetus) [47]. For these stages and more advanced ones, chemotherapy remains the treatment of choice when pregnancy is maintained. The role of staging lymphadenectomy in stages IB3-IIA2-IIB is unclear and therefore not recommended. Elective cesarean delivery is preferred in cases of cervical cancer.

#### 3.1.3. Ovarian Cancer Surgery

Ovarian neoplasms occur in approximately 2% of all pregnancies and are most frequently detected in stage IA following routine ultrasound evaluation [14,48,49].

Most adnexal masses detected during pregnancy are benign (with functional cysts being the most common), and a large proportion of them (70%) are resolved spontaneously by the second trimester. Ca125 is a prognostic marker for ovarian cancer that has limited value during pregnancy due to its variability. On the other hand, lactate dehydrogenase (LDH) remains stable throughout pregnancy and is particularly useful in diagnosing dysgerminomas. For persistent cysts larger than 6–8 cm that exhibit rapid growth or complex features (such as solid components or bilaterality), surgical intervention is typically recommended during the second trimester [14,48,49,50].

Germ cell tumors are the most common type of ovarian neoplasms, with mature ovarian teratomas (dermoid cysts) being the most common histological form. Among malignant germ cell tumors, dysgerminomas are the most frequent, making up about 30% of all malignant ovarian cancers diagnosed during pregnancy, followed by serous cystadenomas [5,13,14].

In cases of early-stage invasive epithelial ovarian cancer, when the patient desires to maintain the pregnancy, the recommended treatment involves performing a unilateral salpingo-oophorectomy, along with peritoneal washings, omentectomy and biopsies of the peritoneum. Additionally, pelvic and para-aortic lymphadenectomy should be considered based on the findings from the frozen section analysis. Furthermore, adjuvant chemotherapy may be required [14,48,50].

Regarding borderline tumors, these atypical proliferative tumors usually show benign behavior when limited to the ovary. In most cases, they are diagnosed histologically as incidental findings during a cesarean section. If suspected during pregnancy, the treatment typically involves surgical intervention, including peritoneal washings, unilateral salpingo-oophorectomy, omentectomy and peritoneal biopsies [50].

When dealing with advanced-stage invasive epithelial ovarian cancer, preserving the pregnancy usually prevents the use of standard treatment, leading to a poor maternal prognosis and making pregnancy termination a reasonable option [14,48,50]. However, if the patient chooses to continue the pregnancy, the recommended approach involves neoadjuvant chemotherapy until fetal maturity is reached (followed by interval debulking surgery after delivery) [10,13,14,48,50].

Germ cell and sex-cord stromal tumors are typically diagnosed at Stage I when found during pregnancy [50]. In these cases, the preferred treatment involves unilateral salpingo-oophorectomy and surgical staging without lymphadenectomy [14]. If the presence of the tumor is confirmed, adjuvant chemotherapy should be administered, with paclitaxel-carboplatin or cisplatin-vinblastine-bleomycin being the most common regimen, similar to the approach for non-pregnant women [10].

Fertility-sparing surgery (FSS) typically involves the preservation of at least the contralateral ovary and the uterus (along with staging surgery). However, there is a lack of data regarding the clinical outcomes for women who undergo cystectomy as a fertility-preserving option. Recently, Kajiyama et al. [51] published the results of a retrospective study involving eight patients with early-stage epithelial ovarian cancer treated with cystectomy as part of FSS. Among the patients, two (one with IA stage and endometrioid histology and the other with IC3 stage and mucinous histology) experienced recurrence, one in the pelvic cavity and the other in both ovaries. Unfortunately, one patient died from the disease. The authors concluded that further research is needed to better assess the feasibility of cystectomy as a fertility-sparing approach in this context. Moreover, in a French multicenter study including 313 patients with stage I borderline ovarian tumors, recurrence rates after cystectomy, unilateral salpingo-oophorectomy and bilateral salpingo-oophorectomy were found to be 30.3%, 11% and 1.7%, respectively [52].

Canlorbe et al. stated that FSS has shown favorable fertility outcomes and does not negatively impact survival in patients with borderline ovarian tumors (BOT). It should be considered for young women wishing to conceive (even if peritoneal implants are found during the initial surgery). In cases of persistent infertility, assisted reproductive technology (ART) may be initiated for patients with stage I BOT, although the number of stimulation cycles should be limited. In the context of epithelial ovarian cancer (EOC), FSS is only appropriate for adequately staged patients with stage IA grade 1 (and possibly grade 2, or low-grade tumors in the current classification) serous, mucinous or endometrioid cancers, and these patients must undergo close gynecologic follow-up. FSS may also be considered for patients with stage IC grade 1 (or low-grade) disease. For women with serous, mucinous or endometrioid high-grade FIGO stage IA or low-grade FIGO stage IC1 or IC2 EOC tumors, a bilateral salpingo-oophorectomy combined with uterine conservation could be performed, allowing for pregnancy via egg donation. Lastly, FSS plays a significant role in the management of non-epithelial ovarian cancers, especially for patients with malignant germ cell tumors [53].

#### 3.1.4. Vulvar Cancer Surgery

The incidence of vulvar cancer during pregnancy remains unclear due to its rarity (only 36 cases were reported worldwide between 1955 and 2014). Additionally, underreporting may contribute to the lack of precise data on the incidence of vulvar cancer in pregnancy [54].

The most common signs of vulvar cancer are vulvar mass, vulvar irritation and pruritis. Once the diagnosis is established, surgical techniques and indications for groin lymphadenectomy should adhere to the same guidelines as those for non-pregnant patients. Inguinal dissection is usually postponed until after delivery to minimize surgical morbidity, with sentinel node dissection being another option, if necessary [10,13].

#### 3.1.5. Colorectal Cancer Surgery

The approach to surgical intervention for colorectal cancer in pregnant women may vary depending on several factors, such as the location of the tumor, its stage of progression, the clinical presentation of the disease and the gestational age of the fetus. When a diagnosis of cancer is made during the early months of pregnancy, clinicians may opt for surgical resection of the tumor (while the pregnancy is ongoing) or for the termination of the pregnancy (followed by subsequent surgical excision of the tumor). In situations where cancer is diagnosed later in the gestational period, it may be appropriate to induce early delivery, provided that the gestational age is deemed adequate for a preterm birth. After delivery, surgical treatment can be pursued. When it comes to rectal cancer, the surgical strategy can be significantly more complicated during the latter stages of pregnancy. Although it is possible for a vaginal delivery to occur, medical professionals typically recommend a cesarean section in these cases to reduce the risk of complications, such as significant bleeding or obstruction of the birth canal (especially when dealing with larger tumors that may complicate the delivery process) [47,55].

#### 3.1.6. Malignant Melanoma Surgery

Careful monitoring of skin changes is essential during pregnancy, as such changes can also occur naturally. Any lesion that cannot be attributed to normal pregnancy-related changes should be thoroughly evaluated. For early-stage melanoma, the treatment of choice is surgical excision, with lymphadenectomy performed only if absolutely necessary. Histological analysis of the placenta is recommended due to the significant potential for metastasis to this site [56,57].

### 3.2. Chemotherapy

Typically, systemic treatments, which encompass chemotherapy, hormone therapy, targeted therapies and immunotherapy, are not recommended during the first trimester. This caution stems from the considerable risk associated with these treatments, which can lead to malformations in up to 20% of cases, as well as the potential for miscarriage. Minimizing delays in systemic cancer treatment is essential whenever feasible.

A short summary of the current guidelines can be found in Table 2 regarding the administration of chemotherapy during pregnancy. Table 3, Table 4 and Table 5 list the most common chemotherapeutic agents used during pregnancy, as well as those that are not recommended and possible modifications that can be made to therapeutic regimens to ensure safety.

There has been some clinical experience with cytarabine in managing leukemia, predominantly in combination with doxorubicin, rather than with daunorubicin or idarubicin. This preference is primarily due to the comparatively poorer toxicity profile associated with the latter agents during pregnancy [74].

As a neoadjuvant treatment, pregnant women diagnosed with cervical cancer may benefit from the use of cisplatin in the second trimester to delay radical hysterectomy. Marnitz S et al. observed significant concentrations of cisplatin in amniotic fluid and cord blood (unlike epirubicin and doxorubicin) in the 7 cases studied. However, all the babies were born healthy by elective cesarean section after 32 weeks of gestation [75]. Additionally, cisplatin has been administered in combination with bleomycin for the treatment of germinal tumors without etoposide, given its considerable hematological toxicity and the likelihood of affecting fetal growth. Furthermore, carboplatin in combination with paclitaxel has been used since the second trimester for the treatment of ovarian cancer [76].

### 3.3. Radiotherapy

The administration of radiotherapy during pregnancy remains a highly debated and complex subject.

Table 6 and Table 7 provide recommendations regarding radiotherapy during pregnancy, as well as a classification of risks by therapeutic doses and trimesters. The differences between the stochastic and deterministic effects of radiation are presented in Table 8.

One of the most concerning deterministic effects during the earliest phase of pregnancy, prior to implantation, is the risk of early embryonic death. Nevertheless, this risk remains extremely low for radiation doses under 100 mGy.

When upper body radiation is necessary, customized shielding solutions should be employed to protect the fetus. These may involve advanced shielding techniques, such as bridge construction or tertiary shield walls, which provide more comprehensive coverage than traditional lead aprons [81]. Despite the protection these methods offer, designing and maintaining proper shielding can be complex due to the fetus’s ongoing growth and positional changes during pregnancy. Consequently, shielding may need to be modified or replaced as the pregnancy progresses [82]. This underscores the importance of proactive and continuous collaboration with radiation oncologists and medical physicists, ensuring that each stage of pregnancy is carefully considered when planning radiation treatments.

Over the last 30 years, significant technological advancements in radiotherapy have revolutionized cancer treatment, leading to the adoption of several cutting-edge techniques. These include three-dimensional conformal radiotherapy (3D-CRT), intensity-modulated radiation therapy (IMRT), volumetric modulated arc therapy (VMAT), stereotactic radiotherapy and proton therapy. These methods aim to deliver highly targeted radiation doses directly to tumors while simultaneously reducing the exposure of surrounding healthy tissues and nearby organs, ultimately enhancing both the precision and tolerability of treatments [83,84,85].

In addition to these advances, the use of cone-beam computed tomography (CBCT) has further improved the accuracy of daily radiation delivery, enabling clinicians to verify positioning and dose distribution with a high degree of precision [86]. IMRT-VMAT, in particular, is effective at confining high-dose exposure to specific tumor sites, though it does carry the disadvantage of spreading low-dose radiation to a larger volume of normal tissue.

Considering these factors, the application of advanced radiotherapy techniques in pregnant patients with cancer introduces an increased likelihood of both short- and long-term risks to fetal health. While these methods offer benefits for tumor control, the potential for fetal harm means that their use must be approached with caution, applied only to carefully selected patients and weighed against safer alternatives whenever possible [77].

### 3.4. Targeted Therapy

Data concerning the administration of targeted therapies during pregnancy are exceedingly limited. Due to the lack of robust evidence, it becomes crucial to consider the distinct properties of various agents that may influence their ability to cross the placenta when evaluating the associated risks to the developing fetus.

Table 9 briefly describes the indications, risks and recommendations for targeted therapies in pregnancy.

### 3.5. Immunotherapy

Presently, there are not enough data regarding the use of immunotherapeutic agents in pregnant women. During pregnancy, the maternal immune system undergoes various adaptations to support tolerance towards the semi-allogenic fetus, primarily mediated by regulatory T cells (Tregs) [108]. This modulation raises concerns about agents that interact with different components of the immune response. Immune checkpoint inhibitors (ICIs) target proteins expressed on Tregs, such as PD-1 and its ligand PD-L1, as well as cytotoxic T-lymphocyte-associated protein 4 (CTLA-4) [109]. These proteins are crucial at the maternal–fetal interface and play an essential role in supporting maternal tolerance to the developing fetus [110].

Evidence suggests that anti-PD-1/PDL-1 antibodies are linked to a higher incidence of abortions in animal models. This is likely attributable to their critical function in establishing immunotolerance during the gestational period [111]. While some isolated cases have been reported where patients received treatment with nivolumab or a combination of nivolumab and ipilimumab, no reliable safety data currently exist to advocate for their use during pregnancy. Furthermore, the use of bevacizumab in pregnant patients is not recommended due to its antiangiogenic effects, even though there are some published cases documenting its use [112]. Limited case reports documenting in utero exposure to ICIs have suggested potential risks, including intrauterine growth restriction (IUGR), placental insufficiency and immune-mediated hypothyroidism in the neonate. On the other hand, no structural malformations have been reported [113,114]. Therefore, while ICIs may be cautiously considered if necessary for maternal health, their use remains controversial.

Immunomodulatory agents that are derivatives of thalidomide—such as lenalidomide and pomalidomide—along with methotrexate, have well-established teratogenic effects and are strictly contraindicated during pregnancy [58,115]. Additionally, chimeric antigen receptor T-cell therapy is firmly prohibited in pregnant patients. Other forms of immunotherapy, including recombinant interleukin-2 and intralesional vaccines like bacille Calmette–Guerin and talimogene laherparepvec, are typically discouraged as well [113].

## 4. Recommendations for Labor and Delivery

The presence of cancer might influence the type and timing of delivery, potentially affecting the newborn. According to a cohort study conducted in Lombardy, Northern Italy, pregnant women with a diagnosis of cancer have an increased risk of labor induction and planned delivery [116]. Moreover, newborns had a higher risk of low birth weight and a low Apgar score at 5 min as a consequence of premature birth. Despite the fact that the preferred mode of delivery in this cohort study was elective cesarean section, the authors highlight that cancer treatment in this population of patients should aim to limit iatrogenic prematurity (the awareness that cancer during pregnancy can be treated has increased over time) [4,116].

A primary indication for cesarean section has been reported in cases of abdominal-pelvic cancer and cervical cancer [117,118]. Furthermore, Esposito G. et al. described elective cesarean section as being more frequent among women with lymphoma than among those with maternal breast or thyroid cancer [116]. Regarding thyroid cancer, they also stated that patients with this type of cancer gave birth at term in most cases.

Donghao et al. reported that maternal cancer during pregnancy was correlated with a higher risk for stillbirth and infant mortality (manly among small for gestational age and preterm newborns) [119]. An increased risk of small for gestational age has been stated by some researchers [119], while others did not support this finding (but they report a higher frequency of small for gestational age following exposure to lymphoma) [116].

Patients diagnosed with intracranial tumors may be advised to opt for an early epidural analgesia combined with an assisted second stage of labor or, alternatively, to undergo cesarean section under general anesthesia. This precaution is motivated by concerns related to the potential increase in intracranial pressure that can result from the Valsalva maneuver during labor [120]. In a similar way, individuals who have bone metastases might receive comparable recommendations to minimize the risk of sustaining long bone fractures during the labor process (which can be a significant concern due to the physical stresses involved) [82]. Furthermore, it is imperative for patients classified as having an increased risk of thrombocytopenia—either as a result of their specific cancer type or the treatment regimen they are undergoing—to have a comprehensive blood workup conducted prior to the onset of labor. This preemptive evaluation is essential to facilitate prompt interventions, if necessary (such as administering platelet transfusions). The target platelet counts necessary to ensure safe delivery methods are established as follows: a minimum of 20 to 30 × 10^9^/L is required for vaginal deliveries, while cesarean sections necessitate a threshold of over 50 × 10^9^/L. For pregnant patients requiring an epidural, it is recommended that the platelet count exceed 80 × 10^9^/L, so the risk of complications will be minimum [121].

This thorough approach ensures that the safety of both the patient and the neonate is prioritized throughout the delivery process, reflecting a comprehensive understanding of the medical implications involved in managing these patients during childbirth.

## 5. Breastfeeding Following Cancer During Pregnancy

Breastfeeding is an experience that strengthens maternal–neonatal bonding [122]. Moreover, lactation boosts maternal self-confidence through skin-to-skin contact with the newborn and the act of nursing [123]. In addition, breastfeeding is known to reduce the risk of certain diseases in mothers, such as ovarian cancer and diabetes. However, the influence of breastfeeding on the risk of subsequent breast cancer is not the same across different population subgroups. For example, Martinez M.E. et al. reported that women of Mexican descent who breastfeed for over 12 months have a predisposition to develop triple-negative breast cancer that is more than double the risk of developing luminal A tumors [124].

Regarding the benefits for the neonate, it has been stated that breastfeeding helps to reduce the risk for developing type 1 diabetes, obesity, asthma and food allergies. Moreover, breastfeeding boosts the immunity of the newborn and regulates the gut microbiome. As an exclusive source of nutrition, breastfeeding is recommended for children up to the age of 6 months [125].

The type of cytotoxic agents, the number of chemotherapy cycles and the gestational age at the beginning of the therapy may impact the ability to breastfeed in patients who received cytotoxic therapy during pregnancy [122]. For example, patients who need to complete their chemotherapy regimen after delivery, or mothers whose previous chemotherapy cycle was not completed at least 3 weeks before, should be discouraged from breastfeeding, despite the fact that the newborn’s toxicity may depend on the amount of milk, the oral bioavailability of the drug or the newborn’s pharmacokinetics [126,127]. Some studies report the transfer of cytotoxic drugs (such as methotrexate, cisplatin, and doxorubicin) into human milk [126]. Imatinib and its metabolites were also found in human milk, but the estimated exposure of the newborn to this agent was around 10% of the therapeutic dose [87]. Moreover, no developmental abnormalities were found after short-term breastfeeding when imatinib was administered, but long-term breastfeeding should still be discouraged in these cases [128,129].

Regarding breast cancer, Cardonick E. et al. reported that only 55% of patients who completed the therapy with chemotherapeutic agents before delivery were able to breastfeed [68]. Moreover, 45% to 63.5% of patients who received antenatal chemotherapy reported a need to supplement their newborns’ feeding due to a reduction in milk production [68,122,130]. Furthermore, higher levels of distress were correlated with reduced milk production [131]. These patients often worry about the safety of breastfeeding. As a result, they should be reassured that breastfeeding is safe, protective, and reduces the risk of cancer recurrence by 41% in comparison with age-matched controls [132].

## 6. Postpartum and Pediatric Considerations

A histological examination of the placenta is essential for detecting potential metastases, particularly in patients diagnosed with melanoma [133]. The discovery of placental metastases calls for a thorough reevaluation of maternal staging, as well as an additional assessment of the newborn’s condition. In the postpartum phase, it is important to assess the safety and practicality of breastfeeding, which should be approached through a collaborative discussion involving various medical specialties. Several factors can complicate a patient’s ability to breastfeed, including a history of breast treatment, a short interval since the last chemotherapy session (specifically, less than three weeks), or the necessity to initiate systemic therapy again after giving birth [58,126,134]. However, there is potential for individualized care, as research shows that levels of taxanes and anthracyclines present in breast milk are remarkably low 2–3 days after administration [135]. It is crucial to provide adequate counseling, particularly considering the societal expectations surrounding breastfeeding, to ensure that patients feel adequately supported in their choices [136]. Moreover, it is advisable to consider the implementation of VTE prophylaxis, including the use of low molecular weight heparin and intermittent pneumatic compression devices, during the postpartum period to enhance patient safety.

In the first few days after birth, it is critical that the neonate undergo a thorough evaluation, which includes a complete blood count, liver function tests, and an assessment of renal function. This comprehensive approach is necessary to rule out the possibility of cytopenia and other toxicities linked to in utero exposure, particularly if the infant is less than three weeks post the last chemotherapy cycle [35]. In instances where placental metastases are confirmed, it becomes imperative to refer the neonate to a pediatric oncologist for a more detailed follow-up, aiming to exclude the risk of fetal metastases [133].

Children who were exposed to platinum-based chemotherapy while still in utero should receive a thorough evaluation for potential auditory dysfunction during their first year, with a follow-up assessment scheduled five years later [64,137]. For those who have been exposed to anthracyclines, it is advisable to perform an echocardiogram within the first year of life and to continue monitoring every three years until they reach early adulthood. This approach is crucial for detecting any signs of potential delayed cardiotoxicity that may develop over time [138].

In the long term, these children require careful monitoring for the development of secondary malignancies as well as neurodevelopmental disorders. Although current studies have a lack of reports regarding the rates among children exposed to chemotherapy in utero, there remains a theoretical risk based on patterns observed in childhood cancer survivors [139]. Additionally, existing literature indicates that children who experienced in utero exposure to chemotherapy demonstrate adequate neurological and psychological development, with follow-up extending to the age of nine years [62,140,141].

Moreover, independent of the specific treatment administered, research has indicated that full-scale IQ scores can be negatively influenced by various factors, including preterm birth, maternal mortality and the educational attainment of the mother [141]. According to another study, children who were followed for up to six years after the death of their mother displayed lower verbal IQ and reduced visuospatial long-term memory scores. There is a theoretical concern regarding the impact of in utero exposure to gonadotoxic agents on fertility in children. However, additional studies with longer follow-up periods are needed to draw definitive conclusions. Currently, there is no evidence to suggest that secondary sexual characteristics are altered in children exposed to cancer treatment while in utero [142].

## 7. Fertility Preservation in Women with Pregnancy-Associated Cancer

It is well known that oncological treatment often impacts reproductive capability [143]. Thus, young women with cancer are frequently concerned about possible fertility issues. The inability to give birth can lead to long-term trauma, especially considering that giving birth is a basic human right [144,145]. According to Schover et al., 76% of fertile women with cancer (who did not have children at the time of diagnosis) wanted to have biological children after recovery [146,147]. Moreover, Partridge et al. reported that 63% of patients who already had one child and up to 20% of women with two or more children expressed concerns regarding infertility as a consequence of cancer treatment [148]. Due to the lack of specific guidelines for fertility counseling and fertility preservation for patients with cancer diagnosed during pregnancy, a large proportion of them do not consult a fertility specialist to discuss these issues before undergoing oncological treatment. As a result, if family planning was not accomplished by these women before starting oncological therapy, a visit to a reproductive medicine specialist should be recommended [149,150,151].

Standard fertility preservation methods (such as cryopreservation of ovarian tissue and cryopreservation of fertilized or unfertilized oocytes) are usually considered not feasible during pregnancy. Furthermore, gonadotoxic treatment may lead to symptoms of hormonal deficiency, mood disorders and sexual dysfunctions, which should be evaluated in a timely manner. After birth, residual reproductive capacity and fertility preservation options should be reevaluated. Hormone replacement therapy should be started at that point (to reduce the short- and long-term effects of premature menopause such as mood changes, sleep disorders and sexual problems) if ovarian reserve is depleted due to cancer treatment and if it is also considered oncologically safe [143,152].

Regarding fertility-sparing surgery, information on this subject has been provided in the Ovarian Cancer Surgery section.

## 8. Emotional and Psychological Effects of Cancer During Pregnancy

Pregnancy is considered a sensible period for women. During this, patients experience a mixture of positive feelings (happiness and enjoyment) and negative feelings (anxiety, stress and adaptive difficulties). Moreover, cancer is considered a life-threatening disease. Patients diagnosed with cancer during pregnancy have their first thoughts towards their infant and the possible consequences associated with this diagnosis. As a result, patients feel emotions of guilt, grief and loss of control [153]. Furthermore, it is well known that pregnant women have a higher risk for mood disorders than patients who do not have cancer [131]. Thus, a standard of care for women with pregnancy-associated cancer is to refer to psychosocial services.

It has been reported that non-pregnant women with a diagnosis of cancer experience high levels of psychosocial distress and post-traumatic stress disorders, anxiety and depression. These high levels were observed during all stages (early phases of diagnosis, the period of cancer treatment, and follow-up) [154]. Similar psychological effects were observed in patients diagnosed with cancer during pregnancy [131]. Decreased levels of concern were correlated with positive coping strategies [155]. According to Vandenbroucke et al., both parents experienced high levels of distress due to many concerns regarding the infant’s health, the cancer diagnosis, the oncological therapy and the outcome of the pregnancy [156]. It has been reported that pregnant nulliparous women showed more concerns than pregnant multiparous women. In general, pregnant patients tended to overestimate the impact of starting chemotherapy on short- and long-term effects on the children. Even more, an oncological disease with a more advanced stage was associated with higher concerns (but not with an increased desire for abortion) [131].

It has been stated that in the postpartum period, mothers who do not have cancer experience a wide range of mental disorders (such as depression) as a result of various factors, including delivery by cesarean section, preterm birth, low birth weight newborns and postpartum anemia [157]. Women diagnosed with cancer during pregnancy often face even more of these risk factors for mental disorders, with preterm birth being the most common (up to 50% of these women will experience iatrogenic-induced preterm delivery) [11]. Additionally, anxiety symptoms are experienced to a greater degree by parents of very low birth weight newborns [158]. Up to 41% of offspring of mothers with cancer during pregnancy are admitted to the neonatal intensive care unit. As a result, anxiety and depression levels are even higher, and both parents should receive psychological counseling [159].

## 9. Conclusions

Caring for patients diagnosed with cancer during pregnancy poses multiple complex medical, ethical, legal and psychosocial challenges. Timely cancer diagnosis in pregnancy requires careful evaluation of symptoms that may overlap with normal pregnancy changes. The inherent difficulty of balancing maternal and fetal well-being is further complicated by the increasing number of treatment options, the rarity of this clinical scenario and the lack of substantial data to inform patients about the short- and long-term risks for themselves and their offspring. Breast cancer is the most common form of cancer in pregnant women. When cancer is detected in a pregnant patient, it is crucial to consider the week of gestation in which the diagnosis is made, as well as the characteristics of the tumor. A multidisciplinary team is strongly recommended to assess the situation and guide the patient and her family through the processes of informing, diagnosis and treatment. Radiation should generally be avoided. However, if clinically justified for the benefit of the mother or fetus, it can be used with adequate protection, taking into account that a delay in diagnosis may pose greater risks for the patient. Chemotherapy may be administered starting at week 14 of gestation, with doxorubicin being the first-line treatment option for breast cancer. Surgical interventions may be performed during pregnancy, but it is essential to adjust anesthesia times accordingly and to manage postoperative pain effectively (as unmanaged pain can lead to contractions that may advance delivery). Cancer treatment during pregnancy should prioritize minimizing iatrogenic prematurity, emphasizing that effective cancer management is increasingly recognized as compatible with pregnancy. Breastfeeding provides essential benefits for both mother and newborn, but its safety after cancer treatment during pregnancy depends on treatment type and timing. Fertility concerns are significant for women with pregnancy-associated cancer, yet standard preservation methods are often unfeasible during pregnancy. Postpartum evaluation and timely fertility counseling are essential to address reproductive capacity and mitigate the effects of gonadotoxic treatments. Cancer during pregnancy amplifies emotional challenges, with heightened risks of anxiety, depression and distress for both parents. Timely psychosocial support is vital to address these concerns and promote coping strategies for better mental health outcomes.

## Figures and Tables

**Table 1:** Imaging techniques and pregnancy.

Imaging Technique	Radiation Type	Safety Considerations	Additional Notes
Ultrasound	Non-ionizing	Considered safe for diagnostic use, without risk of ionizing radiation [15]	Preferred imaging technique, especially in early pregnancy. Use of Doppler ultrasound in the first trimester should be cautious due to potential risks.
MRI (Magnetic Resonance Imaging)	Non-ionizing	Safe when conducted with proper safety measures (magnetic field < 1.5 Tesla) [16]	Non-invasive imaging; no ionizing radiation, but requires adherence to strict protocols for fetal safety.
X-ray	Ionizing	Generally low radiation dose but it should be avoided if possible [15,17,18]	Not recommended, particularly for abdominal or pelvic imaging.
CT Scan (Computed Tomography)	Ionizing	Higher radiation dose compared to X-rays; minimize exposure [17]	Only recommended for specific cases where other imaging techniques are not feasible. Should be avoided for abdominal and pelvic imaging.
Iodinated Contrast Agents	Ionizing	No significant adverse effects observed; potential risk of fetal hypothyroidism [19,20].	Use only if no alternative imaging is available and essential for diagnostic purposes.
Gadolinium-Based Contrast Agents	Ionizing (indirect effect)	Associated with adverse health conditions, including rheumatological disorders, inflammatory responses and infiltrative skin diseases; no conclusive safety data for pregnancy [21].	Not recommended during pregnancy due to potential risks. Should be avoided unless absolutely necessary.

Radiological imaging techniques and contrast agents during pregnancy: safety considerations.

**Table 2:** Chemotherapeutic and pregnancy trimesters.

Time Period	Recommendations
Before 12–14 weeks	Avoid chemotherapy due to high risk of fetal malformation and stillbirth during organogenesis [58,59].
After 12–14 weeks	Many chemotherapeutic agents are considered safe; follow established protocols [11,35,59,60,61,62,63].
Delivery scheduling	Avoid chemotherapy during the hematologic nadir to minimize risks like neutropenia and thrombocytopenia [11,35,58,59,60,61,62,63].
Pre-delivery pause	Discontinue chemotherapy 1–3 weeks prior to spontaneous (38–39 weeks) or planned delivery [64].
Interdisciplinary care	Collaborate with obstetricians, oncologists, neonatologists and psychologists for comprehensive care.

Guidelines for chemotherapy administration during pregnancy.

**Table 3:** Chemotherapeutic agents used during pregnancy.

Agent/Regimen	Safety Profile	Administration Notes
Taxanes (e.g., Paclitaxel)	Safe during the 2nd and 3rd trimesters; low placental transfer; weekly paclitaxel preferred for better toxicity profile [65,66].	Administered weekly or every 3 weeks, depending on protocol and maternal tolerability [65,66].
Anthracyclines Examples: Doxorubicin, Epirubicin, Pegylated/Non-pegylated Liposomal Doxorubicin.	Safe in 2nd and 3rd trimesters; minimal placental transfer [40,67].	Avoid idarubicin due to teratogenic potential; Some studies have reported a tendency for prematurity and low birth weight in some cases [68,69]. In contrast to idarubicin and daunorubicin, both doxorubicin and epirubicin do not cause acute or delayed cardiotoxicity.
FAC/FEC/AC/EC Regimens (includes 5-Fluorouracil, Doxorubicin, Epirubicin and Cyclophosphamide)	Frequently used for breast cancer; safe with no acute/delayed cardiotoxicity [40,67,70].	Requires careful monitoring for neutropenia; supportive care measures like growth factors may be needed [64,71].
CHOP Regimen (includes cyclophosphamide, doxorubicin, vincristine and prednisone).	Used for non-Hodgkin lymphoma; safe in the 2nd and 3rd trimesters [72].	Adjust dose based on maternal weight; consider prophylaxis for tumor lysis syndrome [39,71,73].
ABVD Regimen (includes doxorubicin, bleomycin, vinblastine and dexamethasone).	Used for Hodgkin’s disease; safe during the 2nd and 3rd trimesters [72].	Avoid during 1st trimester; monitor fetal growth and maternal pulmonary function due to bleomycin use [71,73].

Common chemotherapeutic agents used during pregnancy.

**Table 4:** Chemotherapeutic agents and alternatives used during pregnancy.

Agent/Regimen	Reason	Alternatives
Idarubicin	Potential teratogenicity even in the 2nd trimester; low molecular weight and high placental transfer [67,69].	Doxorubicin or epirubicin [40].
BEACOPP Regimen (a combination of bleomycin, etoposide, doxorubicin, cyclophosphamide, vincristine, procarbazine and prednisone)	High dose-intensity; contraindicated due to risk of toxicity [71,73].	CHOP or ABVD for Hodgkin’s and non-Hodgkin lymphoma [71,72].
High-dose alkylating agents Examples: Busulfan, Cyclophosphamide, Melphalan.	Not recommended due to high toxicity [71,73].	Standard-dose cyclophosphamide as part of adjusted regimens (e.g., FAC or CHOP) [39,72].
Methotrexate	Contraindicated due to teratogenic effects and potential harm to the fetus [71,73].	Leucovorin rescue for cases of inadvertent exposure.

Agents/regimens to avoid during pregnancy.

**Table 5:** Peculiarities for chemotherapy in pregnancy.

Clinical Scenario	Recommended Adjustments
Breast cancer in 2nd/3rd trimester	Use anthracycline-based regimens (e.g., FAC or FEC); avoid delivery during nadir; monitor fetal growth [70].
Hematologic malignancies	Use regimens like CHOP for non-Hodgkin lymphoma or ABVD for Hodgkin’s; avoid BEACOPP; dose adjust based on maternal weight [67,71,72].
Soft tissue sarcomas	Doxorubicin + ifosfamide; delay treatment initiation until 3rd trimester if feasible [72,74].
Patient opting to continue pregnancy despite contraindications	Modify protocols to exclude teratogenic agents; consider risk-benefit analysis.

Adjusted treatment strategies for pregnancy.

**Table 6:** Timing of pregnancy and radiotherapy.

Aspect	Recommendations
Timing	Preferably postpone radiotherapy until after delivery, especially for pelvic tumors [77].
Fetal safety	Ensure fetal radiation exposure is under 100 mSv to minimize risks [22].
Stage of pregnancy	Safer in the 1st and early 2nd trimester for upper body radiation due to greater physical separation [78].
Collaboration	Work closely with medical physicists and oncologists for accurate dose calculation and shielding.

General guidelines for radiotherapy during pregnancy.

**Table 7:** Doses of radiation and pregnancy.

Radiation Dose	Trimester	Potential Risks	Notes
<100 mGy	Any trimester	No significant risk to fetal development [22].	Considered safe threshold for most pregnancies.
100–150 mGy	1st and 2nd trimester	Low risk of malformations; risks of microcephaly or mental retardation increase above 150 mGy [77,79].	Organogenesis stage (weeks 2–7) and brain development (weeks 8–15) are critical periods.
>500 mGy	1st trimester	High risk of mental retardation, microcephaly, congenital malformations and early embryonic death [77,79,80].	Deterministic effects observed when dose threshold is exceeded.
100–499 mGy	2nd trimester	Risks include mental retardation (~2% probability), microcephaly, cataracts, sterility, and cancer [77,79,80].	Risks decrease compared to the 1st trimester but remain present.
<500 mGy	3rd trimester	Lower likelihood of severe effects; isolated cases of growth abnormalities and microcephaly have been observed [77,79].	Safest trimester for potential exposure.

Fetal radiation exposure and risks by dose and trimester.

**Table 8:** Biological effects of radiations.

Type of Biological Effect	Characteristics	Dose-Dependence
Stochastic Effects	Occur randomly. No specific threshold [79,80].	Probability of occurrence increases with dose, but severity does not depend on the dose.
Deterministic Effects	Clear cause–effect relationship; occur only when exposure exceeds a specific threshold [79,80].	Both probability and severity increase as radiation doses rise.

Comparison of stochastic and deterministic radiation effects.

**Table 9:** Targeted therapies in pregnancy.

Category	Description	Agent/Therapy	Indication	Risks and Effects	Recommendations
TKIs (Tyrosine Kinase Inhibitors)	Small molecules that may cross the placental barrier.	Imatinib	CML (chronic myeloid leukemia), gastrointestinal tumors	Placental transfer; limited case reports of successful pregnancies without adverse effects after the first trimester.	Avoid; use cautiously if absolutely necessary [58,87,88,89,90].
		Other TKIs	Various oncological indications	Insufficient data; potential teratogenic effects.	Contraindicated [90].
Monoclonal Antibodies	Target specific molecules or receptors, e.g., HER2 (human epidermal growth factor receptor 2) or B lymphocytes.	Trastuzumab	Breast cancer, gastric cancer	Anhydramnios, oligohydramnios.	Contraindicated throughout pregnancy [58,91,92,93,94,95].
		Rituximab	Non-Hodgkin lymphoma, autoimmune diseases	No fetal malformations after the first trimester; Transient neonatal cytopenia.	Cautious use in the second and third trimesters [61,90,96,97,98,99].
Antiangiogenic Agents	Target vascular endothelial growth factor pathways (VEGF).	TKIs and anti-VEGF mAbs (monoclonal antibodies)	Various oncological indications	Teratogenic effects observed in animal studies.	Not recommended during pregnancy [58,90].
ATRA (All-trans Retinoic Acid)	Vitamin A derivative, highly teratogenic, used for APL (acute promyelocytic leukemia) treatment	ATRA (Tretinoin)	APL	Teratogenic in the first trimester; safer in the second and third trimesters according to case reports.	Contraindicated in the first trimester; cautious use in the second and third trimesters for APL management, usually in combination with anthracyclines [58,100,101,102,103].
		Arsenic	APL	Fetal malformations, stillbirth.	Contraindicated in all trimesters [100,102,103,104].
IFN-α (Interferon-α)	Cytokine with minimal placental crossing	IFN-α	Melanoma, hematological cancers	Minimal placental transfer; no fetal malformations reported.	Safe for use throughout all trimesters [58,71,90,105,106,107].

Targeted therapies during pregnancy: indications, risks and recommendations.
